# Effectiveness of symptom-based diagnostic HIV testing versus targeted and blanket provider-initiated testing and counseling among children and adolescents in Cameroon

**DOI:** 10.1371/journal.pone.0214251

**Published:** 2019-05-06

**Authors:** Habakkuk A. Yumo, Rogers A. Ajeh, Marcus Beissner, Jackson N. Ndenkeh, Isidore Sieleunou, Michael R. Jordan, Nadia A. Sam-Agudu, Christopher Kuaban

**Affiliations:** 1 R4D International Foundation, Yaoundé, Cameroon; 2 Center for International Health, Ludwig-Maximilians-Universität of Munich, Munich Germany; 3 Faculty of Health Sciences, University of Buea, Buea, Cameroon; 4 School of Public Health, University of Montreal, Montreal, Canada; 5 Tufts University School of Medicine, Boston, Massachusetts, United States of America; 6 Institute of Human Virology Nigeria, Abuja, Nigeria; 7 Institute of Human Virology and Department of Pediatrics, University of Maryland School of Medicine, Baltimore, United States of America; 8 Faculty of Health Sciences, University of Bamenda, Bamenda, Cameroon; KEMRI Wellcome Trust Research Programme, KENYA

## Abstract

**Objectives:**

The concurrent implementation of targeted (tPITC) and blanket provider-initiated testing and counselling (bPITC) is recommended by the World Health Organization (WHO) for HIV case-finding in generalized HIV epidemics. This study assessed the effectiveness of this intervention compared to symptom-based diagnostic HIV testing (DHT) in terms of HIV testing uptake, case detection and antiretroviral therapy (ART) enrollment among children and adolescents in Cameroon, where estimated HIV prevalence is relatively low at 3.7%.

**Methods:**

In three hospitals where DHT was the standard practice before, tPITC and bPITC were implemented by inviting HIV-positive parents in care at the ART clinics to have their biological children (6 weeks-19 years) tested for HIV (tPITC). Concurrently, at the outpatient departments, similarly-age children/adolescents were systematically offered HIV testing via accompanying parents/guardians. The mean monthly number of children tested for HIV, identified HIV-positive and ART-enrolled were used to compare the outcomes of different HIV testing strategies before and after the intervention.

**Results:**

In comparing DHT to bPITC, there was a significant increase in the mean monthly number of children/adolescents tested for HIV (223.0 vs 348.3, p = 0.0073), but with no significant increase in the mean monthly number of children/adolescents: testing HIV-positive (10.5 vs 9.7, p = 0.7574) and ART- enrolled (7.3 vs 6.3, p = 0.5819). In comparing DHT to tPITC, there was no significant difference in the mean monthly number of children/adolescents: tested for HIV (223 vs 193.8, p = 0.4648); tested HIV-positive (10.5 vs 10.6, p = 0.9544), and ART-enrolled (7.3 vs 5.8, p = 0.4672). When comparing DHT versus bPITC+tPITC, there was a significant increase in the mean monthly number of children/adolescents: tested for HIV (223.0 to 542.2, p<0.0001), testing HIV-positive (10.5 vs 20.3, p = 0.0256), and ART-enrolled (7.3 vs 12.2, p = 0.0388).

**Conclusions:**

These findings suggest that concurrent implementation of bPITC+tPITC was more effective compared to DHT in terms of HIV testing uptake, case detection and ART enrolment. However, considering that DHT and bPITC had comparable outcomes with regards to case detection and ART enrolment, bPITC+tPITC may not be efficient. Thus, this finding does not support concurrent bPITC+tPITC implementation as recommended by WHO. Rather, continued DHT+tPITC could effectively and efficiently accelerate HIV case detection and ART coverage among children and adolescents in Cameroon and similar low-prevalence context.

## Introduction

Over the past decade, remarkable progress has been made in HIV care and treatment for people living with HIV (PLHIV). However, children and adolescents have not had proportionate benefits from life-saving antiretroviral therapy (ART). In 2017, global ART coverage for children less than 15 years old was 52%, compared to 59% in adults [1]. This ART coverage gap was especially alarming in the West and Central Africa Region (WCAR), where only 26% of children younger than 15 years of age [1] and 36% of adolescents (10–19 years) eligible for ART were receiving treatment [2]. West and Central Africa is lagging behind other regions in Sub-Saharan Africa and the world in reaching children and adolescents with HIV treatment [2]. In 2017 for example, the ART coverage in children less than 15 years was at least twice higher in Eastern and Southern Africa compared to Western and Central Africa (59% vs 26%) [1].

Cameroon is a WCAR country with an estimated relatively low HIV prevalence of 3.7% and 470 000 PLHIV, including 40 000 children and 31 000 adolescents [1]. The gap in pediatric HIV treatment is even wider in this country, with only 25% of eligible children below 15 years on ART compared to 51% of adults [1].

Though multiple factors account for current gaps in pediatric and adolescent ART coverage, poorly-implemented and/or ineffective case-finding strategies are major barriers to ART roll-out in this population [3,4,5]. To address these gaps, the World Health Organization (WHO) recommends provider-initiated-testing and counseling (PITC) with two implementation modalities: the universal or routine testing approach [6,7,8,9] and the index case-testing or targeted testing approach [7,8,9]. While the universal testing approach recommends HIV testing for all clients visiting a health facility for any reason, the index case testing model promotes HIV testing among biological children of known HIV-positive parents in care. For the purpose of this study, the universal testing approach is referred to as “blanket provider-initiated-testing and counselling” (bPITC) and the index case-testing approach is described as “target provider-initiated-testing and counselling” (tPITC) [10].

The WHO recommends that in the context of a generalized HIV epidemic, both bPITC and tPITC should be implemented to enhance HIV case-finding and prompt treatment among infants, children and adolescents [6, 7, 8, 9]. Despite this recommendation, the uptake of both strategies remains sub-optimal in most sub-Saharan African countries, including Cameroon where the standard HIV testing practice is the symptom-based HIV testing (DHT) approach. This gap results from inconsistent and fragmented implementation of bPITC and tPITC strategies attributed to many factors, including fear of stigma among parents/caregivers, lack of staff training, lack of HIV testing kits, poor commitment from facility leadership, and missed parental consent to test children [11,12]. These factors are multifaceted barriers operating at the patient, provider, community and national policy levels [13].

Previous studies have reported on outcome of singular bPITC [14,15,16] or tPITC [17,18] approaches among children and adolescents. There is little published information on the effectiveness of concurrent implementation of both strategies as per WHO recommendations, and this paucity of data is likely contributing to poor implementation. Likewise, there is a dearth of literature on the comparative effectiveness of DHT vs bPITC, or DHT vs tPITC. This study aimed to bridge this knowledge gap and provide data to stakeholders to intensify evidence-based HIV case-finding for children and adolescents.

## Materials and methods

### Study design

This was a quasi-experimental pre-and post-intervention study comparing the effectiveness of concurrent implementation of bPITC and tPITC with the symptom-based diagnostic HIV testing (DHT) in terms of HIV testing uptake, case detection and ART initiation at three healthcare facilities in Cameroon. Prior to study implementation (pre-intervention period), tPITC was not implemented at the ART clinics of the study hospitals and DHT was the prevailing HIV testing clinical practice implemented at the outpatient departments (OPDs) of these hospitals. In the post-intervention (post-intervention period), tPITC and bPITC were introduced and concurrently implemented in these health facilities (tPITC at the ART clinics and bPITC at the OPDs).

#### Study period

The study was implemented for a period of 6 months from July through December 2015 at Limbe Regional Hospital (LRH) and from June through November 2016 for Ndop and Abong-Mbang District Hospitals. These periods constituted the post-intervention period of the study. The pre-intervention period corresponded to 6 months prior to study initiation at each facility. Hence, the pre-intervention period was from January through June 2015 for Limbe and from December 2015 through May 2016 for the other two sites.

#### Study population

For both the pre and post-intervention periods, eligible participants were children and adolescents aged 6 weeks-19 years with unknown HIV status. In the pre-intervention period, the study population were children/adolescents presenting at the outpatient department (OPD) at the three hospitals with clinical manifestations of HIV/AIDS. In the post-intervention period, the study population comprised two sub-groups of children/adolescents: 1) biological children/adolescents of HIV-positive parents enrolled in HIV care at ART clinics of the study hospitals and 2) all children/adolescents who were seen for any medical reason at the OPDs of those hospitals.

### Setting and implementation framework

This study was part of the “Active Search for Pediatric HIV/AIDS” (ASPA), a larger study aimed at comparing the acceptability, feasibility and effectiveness of tPITC versus bPITC in Cameroon [10]. This larger study was implemented at three government-owned health facilities previously mentioned. Located in three different regions, they were purposefully selected to ensure inclusion of population of urban (Limbe), semi-urban and rural areas (Ndop: North-West and Abong-Mbang: East). All study facilities provide comprehensive health care services including HIV prevention, diagnosis, care and treatment for adults, children/adolescents and pregnant women. In addition to supports from the government of Cameroon, all HIV services were funded by the Global Fund to Fight HIV/AIDS, TB and Malaria (Global Fund) at all study facilities, and by the US President’s Emergency Plan for AIDS Relief (PEPFAR) at the Limbe and Ndop hospitals.

Before this study, there was no systematic HIV testing for children through their parents (routine index-case testing or tPITC) in care in the three hospitals and no data on this strategy were available at these sites. Likewise, children/adolescents visiting the outpatient departments (OPDs) for consultations were not systematically offer HIV testing. Thus, before the study, there was no implementation of tPITC at the ART clinics nor bPITC at OPDs of the three health facilities. Instead, outpatient HIV testing for children/adolescents at the OPDs was essentially symptom-based and for diagnosis-of-illness purposes. Care providers offered HIV testing to children/adolescents presenting with signs and symptoms suggestive of HIV infection. Thus, the prevailing standard clinical practice for HIV testing during the pre-intervention period was limited only to the diagnostic HIV testing (DHT) [9].

### Intervention

#### Training and supports

Prior to implementation and data collection, the study provided the following inputs to the study sites: staff training on tPITC and bPITC implementation, provision of HIV testing kits (to eliminate stock-outs during the study), standardized data collection and monitoring tools (registers and forms for HIV testing/results, linkage/ART enrolment), and additional human resources to support study implementation [10]. Specifically, three dedicated staff (two data collectors and 1 data manager) were recruited for each study site. One of these three staff was posted at the ART clinic to ensure that all eligible parents in HIV care were counselled and enrolled in the study, and that their children were tested for HIV and that those positive were linked to care (tPITC). The second staff was posted at the OPD to ensure that all children coming for consultation were approached (via their parent/guardian) for recruitment in the study, tested for HIV (regardless of the reasons of consultation), and that positive cases were enrolled in care (bPITC). The third staff was responsible for the overall coordination of site activities, ensuring compliance with the study protocol and data quality. Finally, community health workers (hospital staff) were re-mobilized to ensure counselling, follow up, reminder calls and community/home-based testing for children/adolescents when applicable.

#### tPITC and bPITC implementation

We implemented tPITC at ART clinics by counseling HIV-positive parents in care to have their biological children of unknown HIV status tested for HIV. At the ART clinics, current patients visiting the clinic for drug refills or new patients referred for enrollment in HIV care were approached and counselled by a trained counsellor for enrolment in the study. Consenting eligible parents were invited to enroll their biological children in the study for HIV testing, either in the hospital or at home/community. At the OPDs (bPITC site), all parents/guardians accompanying child(ren) for consultation were also approached and counselled by a trained counsellor for enrolment in the study. Consenting eligible parents were invited to enroll child(ren) in the study for HIV testing. At both ART clinics and OPDs, children/adolescents of consenting parents were study-enrolled, tested for HIV and positive cases initiated on ART as described below.

#### HIV testing, linkage and ART enrolment

For children below 18 months of age, blood specimen was collected using dried blot spot (DBS) kits and shipped to the reference laboratories for HIV testing by PCR techniques. The reference laboratories were the Cameroon Baptist Convention Reference Laboratory in Mutengue (South West Region) for samples from the Limbe Regional Hospital, the Chantal Biya Foundation Reference Laboratory in Yaoundé (Centre Region) for samples from Abong-Mbang District Hospital and the Bamenda Regional Hospital Reference Laboratory (North West) for samples from Ndop District Hospital. For children 18 months and older, they were tested either in the hospital or in the community at the convenience of the parents. In any either case, HIV testing was done using two rapid tests (RT) for HIV antibody namely Alere Determine HIV-1/2 (Paul Hartman, AG Germany) for RT1 and Oraquick (Alere Medical Co Ltd, Japan) for RT2. The HIV antibody rapid tests were conducted following the national HIV testing algorithm as described in the Cameroon’s national guideline on HIV prevention and management [19]: for children/adolescents tested at facility level, both RT1 and RT2 were done by laboratory technicians in the hospitals’ laboratories. For children/adolescents tested in the community, only RT1 was done by trained community health workers. Children/adolescents tested HIV-positive in RT1 were referred to the hospital for confirmation using RT2. The WHO test and treat policy [20] was not established at site level at study initiation, however implementation was scaled up over time. Thus, in some cases, children/adolescents testing HIV-positive were assessed for ART eligibility using the WHO clinical staging and/or baseline laboratory analysis including CD4 count [19]. Eligible children were initiated on ART following Cameroon’s national guideline on HIV prevention and management [19], which were adapted from WHO’s 2013 HIV prevention and management guidelines [8]. In other cases, children testing HIV-positive were promptly enrolled on ART following WHO’s test and treat policy [20].

### Data collection

#### Pre-intervention period (DHT)

We had no contact with participants for this retrospective phase of the study. Rather, we collected retrospectively routine data on DHT implementation from OPDs, laboratory and ART clinic standardized registers. Trained data clerks used a structured form (see S1 Text) to extract from these registers the numbers of children per month: seen at OPDs, tested for HIV, tested HIV positive and initiated on ART.

#### Post-intervention period (tPITC+bPITC)

Data on tPITC and bPITC implementation and respective outcomes were collected prospectively. For that purpose, trained data clerks used structured forms (see supplementary S1 and S2 Texts) to extract routine data from standardized hospital registers at the OPDs, laboratory and ART clinic. In addition, we used a specific study register at the ART clinic to identify children/adolescents of HIV-positive parents in care. For each strategy, we extracted the number per month of children/adolescents: identified for HIV testing, tested for HIV, tested HIV-positive and enrolled on ART.

### Outcome measures and data analysis

The outcome of implementation of each strategy (DHT alone, bPITC alone, tPITC alone, and bPITC + tPITC) was measured using the arithmetic monthly mean of the following variables: 1) number of children/adolescents tested for HIV (HIV testing uptake), 2) number of children/adolescents testing HIV-positive (HIV case detection) and 3) number of HIV-positive children/adolescents enrolled on ART (ART enrollment). Data were entered into a Microsoft Excel sheet and analyzed using STATA 2013 (College Station, TX: StataCorp LP). We calculated the arithmetic means of the aforementioned variables. The number of values for each variable was 6, representing the number of months of implementation of the testing interventions. When applicable, we reported the values of these means as monthly outcomes for the pre-and post-intervention periods. Using Student’s t-test at 5% significant level, we compared these means before and after the intervention to assess for differences.

#### Ethical considerations

For the retrospective study, inform consent from participants was not required as we had no direct contact neither with patients nor with their medical records. More so, we did not collect personal identifiers information for patients, but rather numerical data from hospital registers. For the post-intervention period, participation in the (larger) study was voluntary for both parents and children. Only parents who consented were enrolled and assent was requested from adolescents above 11 years of age. Consent from parents was obtained via signed written consent form. Likewise, assent for adolescents over the age of 11 was obtained through a signed written assent form. The ASPA study received ethical approval from the Cameroon National Ethics Committee (CNERSH), the Ludwig-Maximilians-Universität, Munich (Germany) and the Albert Einstein College of Medicine (NY, U.S.) [10]. In addition, the study received an administrative authorization from the Cameroon Ministry of Public Health.

## Results

### Outcomes of DHT and bPITC implementation in outpatient departments

A total of 5,891 and 4,643 children/adolescents were seen at the OPDs of the three hospitals during the pre-and post-intervention periods, respectively. The mean monthly number of children/adolescents tested for HIV for the three hospitals combined was 223.0 and 348.3 (p = 0.0073) with DHT and bPITC, respectively. The mean monthly number of HIV-positive children/adolescents identified was 10.5 and 9.6 (p = 0.7574) for DHT and bPITC, respectively. The mean monthly number of HIV-positive children/adolescents enrolled on ART was 7.3 and 6.3 (p = 0.5819) for DHT and bPITC, respectively (Table 1).

**Table 1:** Outcomes of 6-month implementation of DHT versus bPITC+tPITC among children/adolescents at three hospitals in Cameroon.

Variables	Pre-intervention (DHT)	Post-intervention (tPITC+bPITC)		
	Monthly Mean	Monthly Mean	P**	% Change
Number of children/adolescents identified for HIV testing at ART clinics through index parents in HIV care	N/A*	471.5	N/A	N/A
Number of children/adolescents seen in consultation at OPDs	981.8	773.8	0.0187	-21.2%
Number of children/adolescents eligible for HIV testing (ART clinics + OPDs)	981.8	1,245.3	0.0339	26.8%
Number of children/adolescents tested for HIV through tPITC at ART clinics	N/A	193.8	N/A	N/A
Number of children/adolescents tested for HIV through DHT (pre-intervention) or bPITC (post-intervention) at OPDs	223.0	348.3	0.0073	56.2%
Number of children/adolescents tested for HIV in hospitals (ART clinics + OPDs)	223.0	542.2	<0.0001	143.1%
Number of children/adolescents testing HIV+ through tPITC at ART clinics	N/A	10.7	N/A	N/A
Number of children/adolescents testing HIV+ through DHT (pre-intervention) or bPITC (post-intervention) at OPDs	10.5	9.7	0.7574	-7.9%
Number of children/adolescents tested HIV+ in hospitals (ART clinics +OPDs)	10.5	20.3	0.0256	93.7%
Number of children/adolescents enrolled on ART through tPITC at ART clinics	N/A	5.8	N/A	N/A
Number of children/adolescents enrolled on ART through DHT (pre-intervention) or bPITC (post-intervention) at OPDs	7.3	6.3	0.5819	-13.6%
Number of children/adolescents enrolled on ART in the hospitals (ART clinics + OPDs)	7.3	12.2	0.0388	65.9%

DHT: diagnostic HIV testing; tPITC: targeted provider-initiated counselling and testing; bPITC: blanket provider-initiated testing and counselling; ART: antiretroviral therapy; OPD: outpatient department;

*Not applicable because activity not implemented during the pre-intervention period.

**p value comparing the monthly mean outcome of pre-intervention vs. post-intervention period.

### Outcomes of tPITC implementation at ART clinics

During the post-intervention period, we identified 2,829 children/adolescents eligible for HIV testing through index HIV-positive parents at ART clinics of the 3 hospitals. Of these 2,829 children/adolescents, 1,163 were tested for HIV, 64 found HIV positive and 35 enrolled on ART (Table 1). The main reasons for failure to test all identified children/adolescents were: the lack of transport fare to bring children to the hospital, children not living with parents and the lack of time to bring children to the hospital [10]. In comparing DHT outcomes against tPITC: the mean monthly number of children/adolescents tested for HIV was 223 against 193.8 (p = 0.4648); the mean monthly number of children/adolescents tested HIV+ was 10.5 against 10.6 (p = 0.9544), and the mean monthly number of HIV+ children/adolescents enrolled on ART was 7.3 against 5.8 (p = 0.4672), respectively.

### Outcomes of concurrent tPITC and bPITC implementation

**Fig 1:**
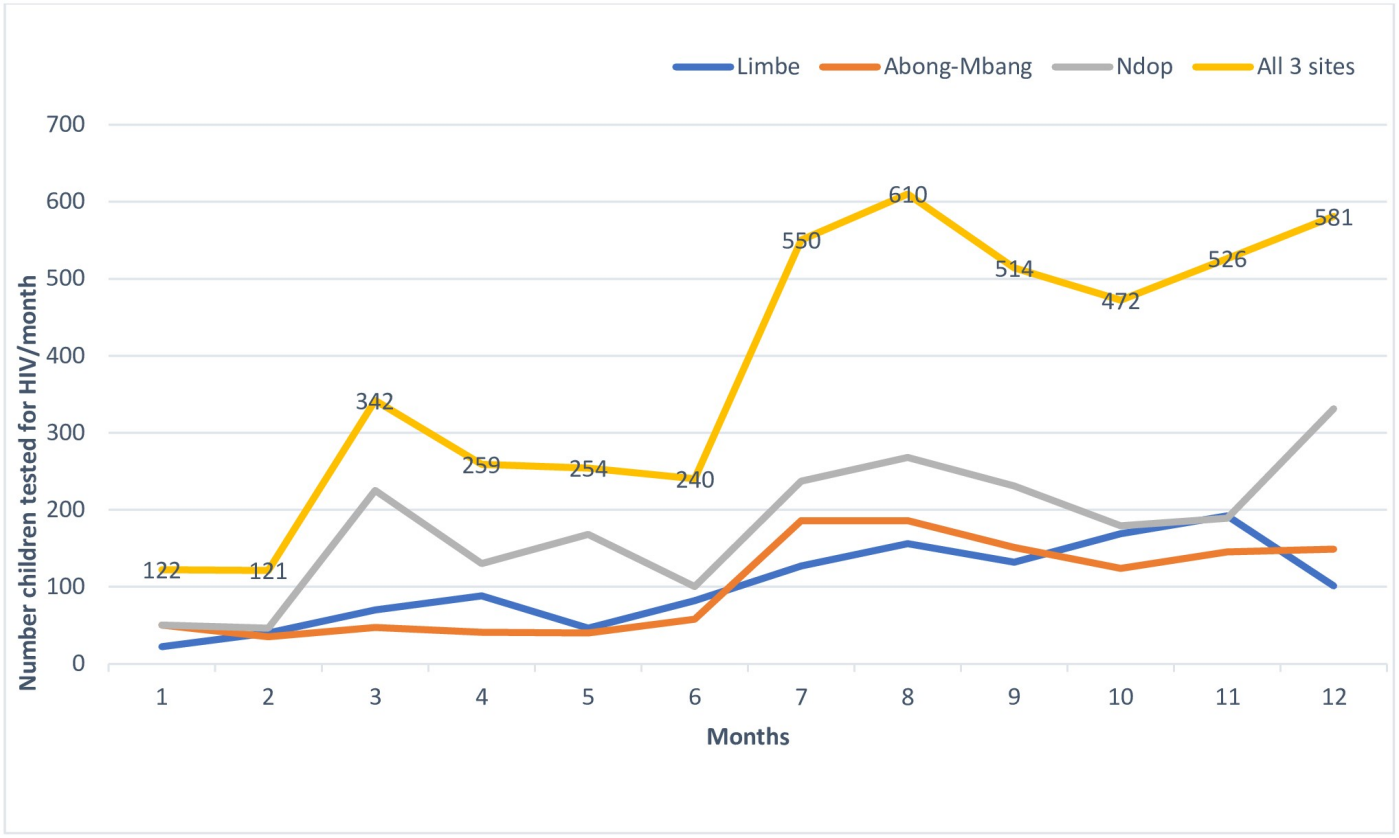
Trends in the number of children/adolescents tested for HIV before and after the intervention.

During the post-intervention period, a total 7,472 eligible children/adolescents were identified for HIV testing in the 3 hospitals including: 2,829 from ART clinics and 4,643 from OPDs. This represents a 27% increase compared to the 5,891 children/adolescents who consulted at the outpatient departments in the pre-intervention period. The mean monthly number of children/adolescents tested for HIV was 223.0 and 542.2 during the pre-and post-intervention periods, respectively. This difference was statistically significant (p<0.0001) and represented an increase of 143% in HIV testing uptake in the three hospitals combined (Fig 1).

**Fig 2:**
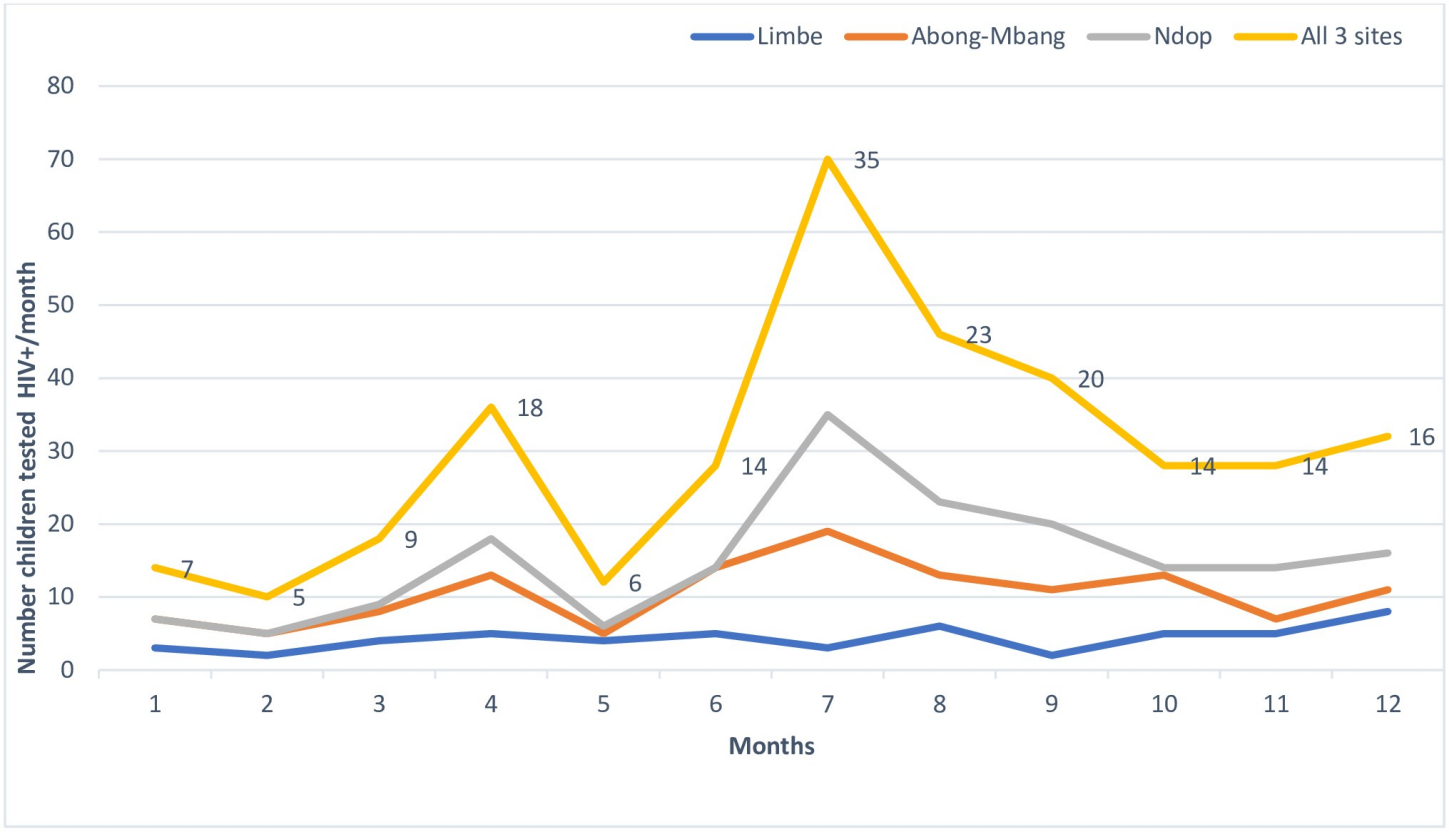
Trends in the number of children/adolescents tested HIV+ before and after the intervention.

The mean monthly number of HIV-positive children/adolescents identified was 10.5 and 20.3 during the pre-and post-intervention period, respectively. This difference was statistically significant (p = 0.0256) and represented a 94% increase of HIV case-detection (Fig 2).

**Fig 3:**
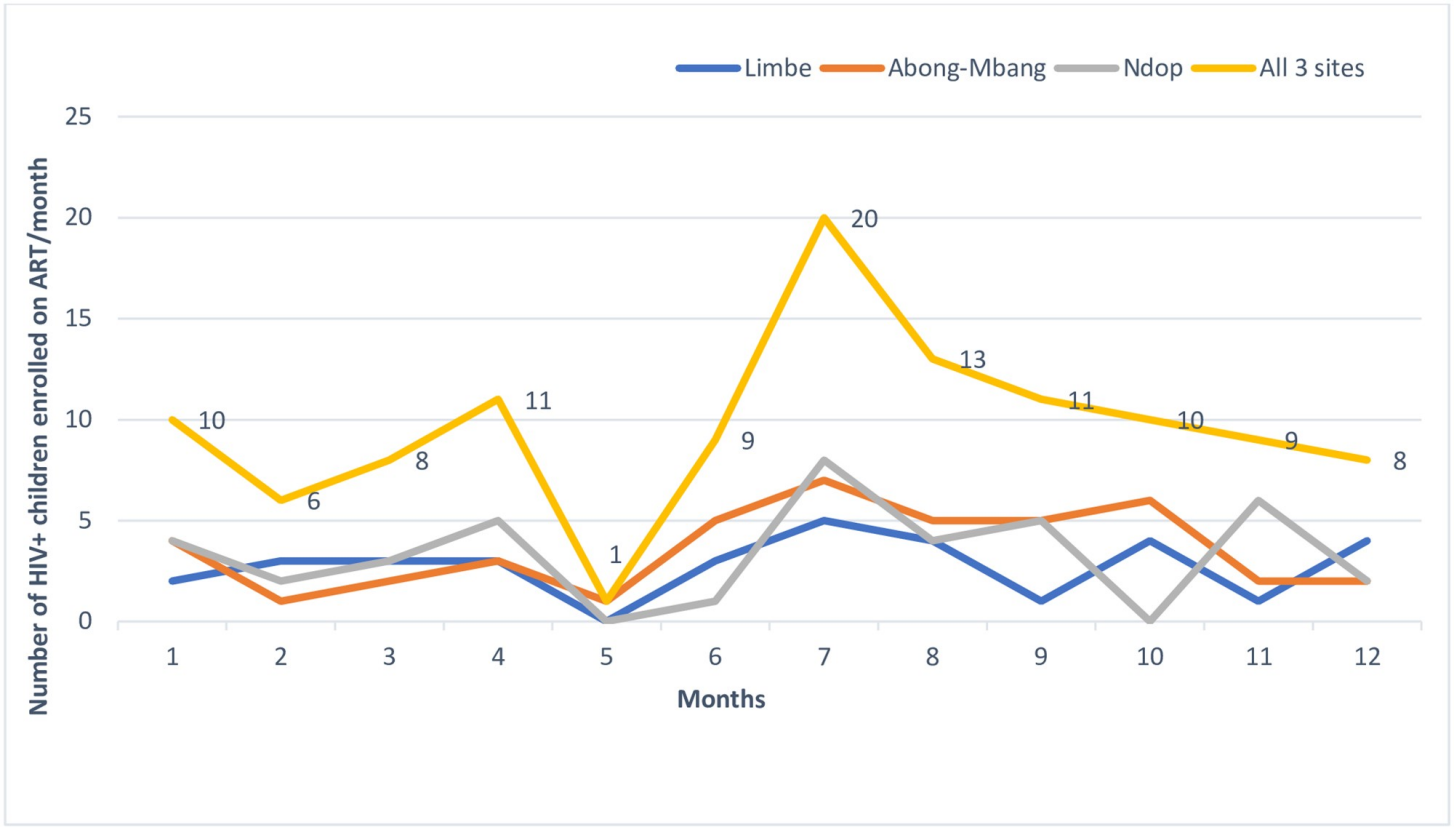
Trends in the number of HIV+ children/adolescents enrolled on ART before and after the intervention.

The mean monthly number of HIV-positive children/adolescents enrolled on ART was 7.3 and 12.2 during the pre-and post-intervention periods, respectively (p = 0.0388), and representing a 66% increase in ART enrolment for the three hospitals combined (Fig 3).

## Discussion

Our study provides previously limited data on the effectiveness of single and concurrent implementation of bPITC and tPITC vs. symptom-based diagnostic HIV testing (DHT) in terms of HIV testing, HIV case detection and ART enrolment among children and adolescents. Implementation of bPITC alone resulted in a 1.5-fold increase in HIV testing uptake compared to DHT. However, bPITC did not improve HIV case detection (10.5 vs 9.6, p = 0.7574) nor ART enrolment (7.3 vs 6.3, p = 0.5819). This could be explained by the fact that during the pre-intervention period, most children tested for HIV were symptomatic; thus, there was a high pre-test likelihood of HIV case-detection given the clinical status of children being tested. This finding does not support bPITC implementation as recommended by WHO. It also suggests that bPITC implementation may not be effective in relatively low HIV prevalence settings such as Cameroon and much of the rest of the WCAR. Previous studies [15, 21] have reported bPITC yield among Cameroonian children, but did not compare this yield with that of DHT (prevailing clinical HIV testing practice). Ferrand et al. reported that bPITC had a higher HIV case detection rate compared to the prevailing clinical practice (mostly DHT) in Zimbabwe [14]. However, the 13.3%, estimated HIV prevalence in Zimbabwe is over 3 times higher than Cameroon’s 3.7% [1]. Thus, our study findings on bPITC are not comparable with available evidence from much- higher prevalence settings. Silvestri et al. and Kayigamba et al. demonstrated that bPITC had comparable case-detection rates with the prevailing clinical practice (mostly DHT) among adults in Zambia [22] (HIV prevalence 11.5%) and Rwanda [23] (HIV prevalence 2.7%), respectively. Though these studies were among adult PLHIV and one in a high-prevalence setting, their results corroborate that of our study and support the need for additional studies to further investigate bPITC effectiveness in comparison with DHT with regards to case detection among children/adolescents in both low and high HIV prevalence settings. The focus of most studies on bPITC implementation outcome among children/adolescents [15,16, 21, 24, 25, 26, 27, 28] has been on implementation and yield; there is scarce comparative data on bPITC versus the prevailing clinical practice in the field or an alternative testing approach as we did in this study. There is need to reduce this evidence gap across high pediatric-HIV burden countries to guide policy and inform effective programming.

During the post-intervention period, the concurrent implementation of tPITC and bPITC resulted in significant increases in HIV testing uptake (223.0 vs 542.2, p<0.0001), case detection (10.5 vs 20.3, p = 0.0256) and ART enrolment (7.3 vs 12.2, p = 0.0388). However, as indicated above, bPITC implementation had comparable case detection and ART enrolment with DHT. Similarly, tPITC had comparable outcomes to DHT in terms of HIV testing uptake, case detection and ART enrolment. Thus, the significant increase observed in HIV testing uptake, case detection and ART enrolment was attributable to the effect of both bPITC and tPITC. However, considering that bPITC had comparable outcomes to DHT with regards to case detection and ART enrolment, the concurrent implementation of bPITC+tPITC may not be efficient in low HIV prevalence settings.

It’s noteworthy mentioning that in a previous article, we demonstrated the superiority of tPITC over bPITC in terms of case detection timeliness among children and adolescents [10]. Similarly, we expect tPITC to have an advantage over DHT in terms of earlier HIV case detection in these sub-populations. Also, it’s important to note the inadequate ART enrolment rate of 54.6% (35/64) with the implementation of tPITC. This figure represents average ART enrolment for all 3 ART clinics. Specifically, ART enrolment was 20% (1/5) at Limbe Regional Hospital, 59.3% (16/27) at Ndop District Hospital and 72.2% (13/18) at Abong-Mbang District Hospital. The higher enrolment rates at Ndop and Abong-Mbang District Hospitals was likely due to the progressive implementation of the WHO “test and treat” policy in these two hospitals during the intervention period. The study ended in Limbe in December 2015 when this policy was not yet effective in Cameroon. We started the study in Abong-Mbang and Ndop in June 2016 and ended in December 2016. The better ART linkage rates could be the results of the longer exposure to the “test and treat” policy in these 2 health facilities. This is an indication of the positive impact of policy change for early ART initiation as evidenced by previous studies [29,30].

### Policy implications

Our study found that compared to DHT, concurrent implementation of bPITC and tPITC was effective in increasing HIV testing uptake, case detection and ART enrolment among children 6 weeks to 19 years of age. However, compared to DHT, bPITC alone was effective in increasing HIV testing uptake, but did not demonstrate superiority with respect to case detection and ART enrolment. These findings do not support the concurrent implementation of bPITC and tPITC as recommended by WHO from 2010 to date [7, 8,9] and this for efficiency reasons. Rather, it suggests that in low HIV-prevalence settings such as Cameroon, the concurrent implementation of DHT and tPITC could be prioritized as an effective and efficient intervention for HIV case-finding and linkage of infected infants, children and adolescents to HIV treatment and care. This is in line with the current United States’ President’s Emergency Plan for AIDS Relief (PEPFAR) Country Operational Plan Technical Guidance for supported countries [31]. PEPFAR’s current guidance does not support bPITC but rather recommends targeted HIV testing at OPDs using a screening tool such as the Bandason tool [32], and reserving universal testing only among high-HIV risk children such as biological children of known PLHIV, children visiting tuberculosis clinics, and those on inpatient medical ward admission [31]. This PEPFAR guidance is applicable to all countries irrespective of HIV prevalence level. However, it may lead to missed opportunities in high HIV-prevalence settings where bPITC yield may be higher. For example, in Zimbabwe, Ferrand et al. reported an increase in HIV case detection from 2.9% to 4.5% among children and adolescents with the implementation of bPITC compared to the prevailing clinical practice (mostly DHT) [12]. Therefore, there is need for further studies to ascertain the outcome of bPITC vs DHT in high HIV prevalence settings. There is also need to assess the outcome of concurrent implementation bPITC and tPITC in these high HIV prevalence countries, especially in Eastern and Southern Africa to inform future guidelines and prioritization of HIV testing strategies for children and adolescents.

### Strengths and limitations

The WHO declares that “efforts in the global research agenda on pediatric HIV must be focused on generating targeted evidence that improves HIV programme implementation through a better understanding of what works for infants and children” [33]. Our study is a response to this call, and provides evidence suggesting that in relatively low HIV prevalence settings such as Cameroon, the concurrent implementation of DHT and tPITC could be more efficient compared to the concurrent implementation of bPITC and tPITC for case detection and ART enrolment among children and adolescents.

Our study however has some limitations. We did not assess the effectiveness of bPITC (universal HIV testing) in other potentially high yield HIV case detection entry points such as TB clinics, malnutrition units and inpatient wards. Thus, our conclusions with regards to the outcomes of bPITC are limited to a single-entry point, which is the outpatient department (OPD). This limitation may be addressed by further studies. In the interim, the utility of bPITC (universal testing) in these potentially high yield service delivery points should be continued as per WHO recommendations. We had limited control over the quality of the retrospective data collected in the pre-intervention period. Thus, our results may have been affected by validity threats inherent to retrospective studies [34]. Additionally, because we used a quasi-experimental design (non-randomized pretest and posttest design), the validity of our results may have been affected by confounding variables and other potential biases intrinsic to non-randomized trials [35] and pre-post design studies [36]. However, it is established that the results of high quality non-randomized control trials can approximate those found in randomized control trials [37] and our study design was similar to that of previous PITC effectiveness studies [14, 22, 23]. To increase the validity of our results, we conducted the study in 3 hospitals found in 3 different geographic locations in Cameroon (South-West, North-West and East Regions).

That notwithstanding, our study could have been affected by the seasonality bias resulting from the natural fluctuation in the number of children visiting the hospitals over time. However, our results show that fewer children were seen at the OPDs during the post-intervention period (S1 Table). Thus, the number of HIV-positive children detected during the intervention could not have been the result of the increase in service utilization usually associated with seasonality bias [38].

### Conclusions and recommendations

Globally, children and adolescents living with HIV are at risk of significant morbidity and mortality if left undiagnosed and untreated. Ironically, WCAR’s low HIV-prevalence countries are lagging behind high-HIV prevalence Eastern/southern African countries in ART coverage for children and adolescents. Poor ART coverage for these sub-populations will not appreciably improve without increased case-detection. However, it is clear that differentiated approaches need to be tailored to the context, including HIV prevalence and pre-test likelihood of HIV infection. These tailored approaches are needed not only to increase yield, but also for efficient use of already-limited and scarce resources.

For both effectiveness and efficiency reasons, we recommend continued symptom-based HIV testing combined with scale-up of tPITC approaches for Cameroon and potentially other low HIV prevalence countries such as in West and Central Africa Region. For higher HIV prevalence countries such as in Eastern/southern Africa Region, we recommend further studies to ascertain the comparative effectiveness of DHT vs bPITC as well as the concurrent implementation of bPITC and tPITC. This evidence is needed to guide the tailoring testing and treatment approaches consistent with settings and subpopulations characteristics. Thus, ensuring that the agenda to End AIDs by 2030 will leave no children behind regardless of where they live.

## Supporting information

S1 Text Structured Form.OPDs. (DOCX)

S2 Text Structured Form.ART Clinics. (DOCX)

S1 Table Outcomes of 6-month implementation of DHT versus bPITC+tPITC. (DOCX)

S1 Dataset Study dataset. (XLSX)

## References

[1] UNAIDS. UNAIDS Data 2018. Geneva: UNAIDS; 2018 http://www.unaids.org/sites/default/files/media_asset/unaids-data-2018_en.pdf. Accessed 27 October 2018.

[2] UNICEF and UNAIDS. Step Up the Pace: Towards an AIDS-free generation in West and Central Africa, UNICEF West and Central Africa Regional Office and UNAIDS Regional Support Team for West and Central Africa. Dakar: UNICEF & UNAIDS; 2017 https://www.unicef.org/publications/files/Step_Up_the_Pace_West_and_Central_Africa.pdf. Accessed 27 October 2018.

[3] Sam-Agudu NA, Folayan MO, Echezona E. Ezeanolue EE. Seeking wider access to HIV testing for adolescents in sub-Saharan Africa. Pediatric Research 2016;79:6 10.1038/pr.2016.28 26882367

[4] Msellati P. Ateba Ndongo F., Hejoaka F., Nacro B. Impediments to HIV testing in HIV-infected children and teenagers in Africa: look for them where they are!. Medecine et Santé Tropicales 2016; 26: 10–14: 26986242 10.1684/mst.2015.0519

[5] Eba PM, Lim H. Reviewing independent access to HIV testing, counselling and treatment for adolescents in HIV-specific laws in sub-Saharan Africa: implications for the HIV response. Journal of the International AIDS Society 2017;20:21456 10.7448/IAS.20.1.21456. 28799324 PMC5577701

[6] WHO. Guidance on provider-initiated HIV testing and counselling in health facilities. Geneva: WHO; 2007 http://apps.who.int/iris/bitstream/10665/43688/1/9789241595568_eng.pdf. Accessed 07 September 2018.

[7] WHO and UNICEF. Policy Requirements for HIV testing and Counselling of Infants and Young Children in Health Facilities. Geneva: WHO and UNICEF; 2010 http://apps.who.int/iris/bitstream/10665/44276/1/9789241599092_eng.pdf. Accessed 07 Sept 2018.

[8] WHO. Consolidated guidelines on the use of antiretroviral drugs for treating and preventing HIV infection: recommendations for a public health approach. Geneva: WHO;2013 http://www.who.int/hiv/pub/arv/arv-2016/en/. Accessed 27 October 2018. 24716260

[9] WHO. Consolidated guidelines on HIV testing services: 5Cs: consent, confidentiality, counselling, correct results, and connection. Geneva: WHO; 2015 http://www.ncbi.nlm.nih.gov/books/NBK316021. Accessed 27 October 2018. 26378328

[10] Yumo HA, Kuaban C, Ajeh RA, Nji AM, Nash D, Anastos K, et al Active case finding: comparison of the acceptability, feasibility and effectiveness of targeted versus blanket provider-initiated testing and counseling of HIV among children and adolescents in Cameroon. BMC Pediatrics 2018;18:309 10.1186/s12887-018-1276-7. 30253758 PMC6156944

[11] Ahmed S, Kim MH, Sugandhi N, Phelps BR, Sabelli R, Diallo MO, et al Beyond early infant diagnosis: case finding strategies for identification of HIV-infected infants and children. AIDS Lond Engl. 2013;27(0 2):S235–45. 10.1097/QAD.0000000000000099. PMC4122794 24361633

[12] Leon N, Lewin S, Mathews C. Implementing a provider-initiated testing and counselling (PITC) intervention in Cape town, South Africa: a process evaluation using the normalisation process model. Implementation Science. 2013;8:97 10.1186/1748-5908-8-97. 23972055 PMC3765808

[13] Davies M-A, Kalk E. Provider-initiated HIV testing and counselling for children. PLoS Med. 2014;11(5):e1001650 10.1371/journal.pmed.1001650. 24866364 PMC4035263

[14] Ferrand RA, Meghji J, Kidia K, Ethel Dauya E, Bandason T, Hilda Mujuru, et al The effectiveness of routine opt-out HIV testing for children in Harare, Zimbabwe. J Acquir Immune Defic Syndr 2016;71:e24–e29. 10.1097/QAI.0000000000000867 26473799 PMC4679347

[15] Zoufaly A, Hammerl R, Sunjoh F, Jochum J, Nassimi N, Awasom C, et al High HIV prevalence among children presenting for general consultation in rural Cameroon. Int J STD AIDS. 2014;25(10):742–4. 10.1177/0956462413518762. 24469969

[16] Govindasamy D, Ferrand RA, Wilmore MSS, Ford N, Ahmed S, Afnan-Holmes H, et al Uptake and yield of HIV testing and counselling among children and adolescents in sub-Saharan Africa: a systematic review. JAIS 2015, 18:20182 10.7448/IAS.18.1.20182. PMC4607700 26471265

[17] Ahmed S, Sabelli RA, Simon K, Rosenberg NE, Kavuta E, Harawa M, et al Index case finding facilitates identification and linkage to care of children and young persons living with HIV/AIDS in Malawi. Tropical Med Int Health. 2017;22(8):1021–9. 10.1111/tmi.12900. PMC5575466 28544728

[18] Wagner AD, Mugo C, Njuguna IN, Maleche-obimbo E, Sherr K, Inwani IW, et al Implementation and operational research: active referral of children of HIV-positive adults reveals high prevalence of undiagnosed HIV. J Acquir Immune Defic Syndr. 2016;73(5):e83–9. 10.1097/QAI.0000000000001184. 27846074 PMC5175406

[19] Republic of Cameroon. Ministry of Public Health. National Guideline on the Prevention and Management of HIV in Cameroon. Yaoundé: Ministry of Public Health;2015 https://aidsfree.usaid.gov/sites/default/files/cameroon_art_2015.pdf. Accessed 30 October 2018.

[20] WHO. Guideline on when to start antiretroviral therapy and on pre-exposure prophylaxis for HIV. Geneva:WHO;2015 http://www.who.int/hiv/pub/guidelines/earlyrelease-arv/en/. Accessed 30 October 2018. 26598776

[21] Penda CI, Moukoko CEE, Koum DK, Fokam J, Meyong CAZ, Talla S, et al Feasibility and utility of active case finding of HIV-infected children and adolescents by provider-initiated testing and counselling: evidence from the Laquintinie hospital in Douala, Cameroon. BMC Pediatrics 2018;18:259 10.1186/s12887-018-1235-3. 30075712 PMC6090739

[22] Silvestri DM, Modjarrad K, Blevins ML, Halale E, Vermund SH, et al A comparison of HIV detection rates using routine opt-out provider-initiated HIV testing and counseling versus a standard of care approach in a rural African setting. J Acquir Immune Defic Syndr. 2011; 56(1): e9–32. 21189483 10.1097/qai.0b013e3181fdb629 PMC3016940

[23] Kayigamba FR, Van Santen D, Bakker MI, Lammers J, Mugisha V, Bagiruwigize E, et al Does provider-initiated HIV testing and counselling lead to higher HIV testing rate and HIV case finding in Rwandan clinics? BMC Infectious Diseases 2016;16:26 10.1186/s12879-016-1355-z 26809448 PMC4727293

[24] Mutanga JN, Raymond J, Towle MS, Mutembo S, Fubisha RC, Lule F, et al Institutionalizing Provider-Initiated HIV Testing and Counselling for Children: An Observational Case Study from Zambia. PLoS ONE 2012;7(4): e29656 10.1371/journal.pone.0029656 22536311 PMC3335043

[25] Kranzer K, Meghji J, Bandason T, Dauya E, Mungofa S, Busza J, et al Barriers to Provider-Initiated Testing and Counselling for Children in a High HIV Prevalence Setting: A Mixed Methods Study. PLoS Med. 2014 5 27;11(5):e1001649 10.1371/journal.pmed.1001649 24866209 PMC4035250

[26] Leon N, Naidoo P, Mathews C. The impact of provider-initiated (opt-out) HIV testing and counseling of patients with sexually transmitted infection in Cape Town, South Africa: a controlled trial. Implement Sci. 2010;5(1):8. 20205841 10.1186/1748-5908-5-8 PMC2825497

[27] Rollins N, Mzolo S, Moodley T, van Rooyen H. Universal HIV testing of infants at immunization clinics: an acceptable and feasible approach for early infant diagnosis in high HIV prevalence settings. AIDS. 2009;23(14):1851–7. 10.1097/QAD.0b013e32832d84fd 19491653

[28] McCollum ED, Preidis GA, Golitko CL, Siwande LD, Mwansambo C, Kazembe PN, et al Routine inpatient human immunodeficiency virus testing system increases access to pediatric human immunodeficiency virus care in sub-Saharan Africa. Pediatr Infect Dis J. 2011;30(5):e75–81. 10.1097/INF.0b013e3182103f8a 21297520 PMC4157210

[29] Tymejczyk O, Brazier E, Yiannoutsos C, Wools-Kaloustian K, Althoff K, Crabtree-Ramı´rez B, et al HIV treatment eligibility expansion and timely antiretroviral treatment initiation following enrollment in HIV care: A metaregression analysis of programmatic data from 22 countries. PLoS Med 15(3): e1002534 10.1371/journal.pmed.1002534. 29570723 PMC5865713

[30] Panayidou Klea, Davies Mary-Ann, Anderegg Nanina, Egger Matthias, The IeDEA, COHERE, PHACS and IMPAACT Collaborations Writing Group. Global temporal changes in the proportion of children with advanced disease at the start of combination antiretroviral therapy in an era of changing criteria for treatment initiation. Journal of the International AIDS Society 2018, 21:e25200 10.1002/jia2.25200. 30614622 PMC6275813

[31] United States President’s Emergency Fund for AIDS Relief (PEPFAR). PEPFAR 2018 Country Operational Plan Guidance for Standard Process Countries. https://www.pepfar.gov/documents/organization/276459.pdf. Accessed 1 November 2018.

[32] Bandason T, McHugh G, Dauya E, Mungofab S, Munyatia SM, Weissc HA, et al Validation of a screening tool to identify older children living with HIV in primary care facilities in high HIV prevalence settings. AIDS 2016, 30:779–785. 10.1097/QAD.0000000000000959 26588175 PMC4937807

[33] WHO. Research for an AIDS Free Generation: A global research agenda for paediatric HIV. Geneva:WHO;2017 http://www.who.int/hiv/pub/toolkits/cipher-research-paediatric-hiv/en/. Accessed 1 November 2018.

[34] Cindy T. Threats to Validity in Retrospective Studies. J Adv Pract Oncol. 2012; 3(3): 181–183. 25031944 PMC4093311

[35] Sterne JAC, Hernán MA, Reeves BC, Savović J, Nancy D, Berkman ND, Viswanathan M, et al ROBINS-I: a tool for assessing risk of bias in non-randomised studies of interventions. BMJ 2016; 355 10.1136/bmj.i4919. PMC5062054 27733354

[36] Marsden E, Torgerson CJ. Single group, pre- and post-test research designs: Some methodological concerns, Oxford Review of Education 2012;38:5, 583–616, 10.1080/03054985.2012.731208

[37] Ferriter M, Huband N. Does the non-randomized controlled study have a place in the systematic review? A pilot study. Crim Behav Ment Health. 2005;15(2):111–20. 10.1002/cbm.43 16470505

[38] Mylene L. How to do (or not to do) … Assessing the impact of a policy change with routine longitudinal data. Health Policy and Planning 2012;27:76–83. 10.1093/heapol/czr004 21278077

