# Supplementary material for: Effectiveness of symptom-based diagnostic HIV testing versus targeted and blanket provider-initiated testing and counseling among children and adolescents in Cameroon
Source: PLoS One. 2019 May 6;14(5):e0214251. doi: 10.1371/journal.pone.0214251 (PMC6502453; doi:10.1371/journal.pone.0214251)
Supplement: S1 Text — (DOCX) [file pone.0214251.s001.docx]

**ASPA Study: Routine Data Form, OPD* (DHT or bPITC)**

Pediatric HIV testing, care and treatment

Health Facility:…………………………………………………………

|  |  | **Jan** | **Feb** | **March** | **April** | **May** | **June** | **July** | **Aug** | **Sept** | **Oct** | **Nov** | **Dec** | **Total** |
| --- | --- | --- | --- | --- | --- | --- | --- | --- | --- | --- | --- | --- | --- | --- |
| Children visiting the OPD | 2014 |  |  |  |  |  |  |  |  |  |  |  |  |  |
|  | 2015 |  |  |  |  |  |  |  |  |  |  |  |  |  |
|  | 2016 |  |  |  |  |  |  |  |  |  |  |  |  |  |
| Children tested for HIV | *2014* |  |  |  |  |  |  |  |  |  |  |  |  |  |
|  | *2015* |  |  |  |  |  |  |  |  |  |  |  |  |  |
|  | *2016* |  |  |  |  |  |  |  |  |  |  |  |  |  |
| Children tested HIV+ | *2014* |  |  |  |  |  |  |  |  |  |  |  |  |  |
|  | *2015* |  |  |  |  |  |  |  |  |  |  |  |  |  |
|  | *2016* |  |  |  |  |  |  |  |  |  |  |  |  |  |
| HIV+ children linked to care | *2014* |  |  |  |  |  |  |  |  |  |  |  |  |  |
|  | *2015* |  |  |  |  |  |  |  |  |  |  |  |  |  |
|  | *2016* |  |  |  |  |  |  |  |  |  |  |  |  |  |
| HIV+ children initiated on ART | *2014* |  |  |  |  |  |  |  |  |  |  |  |  |  |
|  | *2015* |  |  |  |  |  |  |  |  |  |  |  |  |  |
|  | *2016* |  |  |  |  |  |  |  |  |  |  |  |  |  |

*Source: Registers: OPD Consultations, Laboratory, Pre-ART and ART*

**Outpatient Department*
