# Supplementary material for: Effectiveness of symptom-based diagnostic HIV testing versus targeted and blanket provider-initiated testing and counseling among children and adolescents in Cameroon
Source: PLoS One. 2019 May 6;14(5):e0214251. doi: 10.1371/journal.pone.0214251 (PMC6502453; doi:10.1371/journal.pone.0214251)
Supplement: S1 Table — (DOCX) [file pone.0214251.s003.docx]

**Table 1**: **Outcomes of 6-month Implementation of DHT versus bPITC+tPITC among Children/Adolescents at Three Hospitals in Cameroon**

| Variables | Pre-intervention  (DHT) | Post-intervention (tPITC+bPITC) |  | |
| --- | --- | --- | --- | --- |
|  | **Monthly Mean** | **Monthly Mean** | **P**** | **% Change** |
| Number of children/adolescents identified for HIV testing at ART clinics through index parents in HIV care | N/A* | 471.5 | N/A | N/A |
| Number of children/adolescents seen in consultation at OPDs | 981.8 | 773.8 | 0.0187 | -21.2% |
| Number of children/adolescents eligible for HIV testing (ART clinics + OPDs) | 981.8 | 1,245.3 | 0.0339 | 26.8% |
| Number of children/adolescents tested for HIV through tPITC at ART clinics | N/A | 193.8 | N/A | N/A |
| Number of children/adolescents tested for HIV through DHT (pre-intervention) or bPITC (post-intervention) at OPDs | 223.0 | 348.3 | 0.0073 | 56.2% |
| Number of children/adolescents tested for HIV in hospitals (ART clinics + OPDs) | 223.0 | 542.2 | <0.0001 | 143.1% |
| Number of children/adolescents testing HIV+ through tPITC at ART clinics | N/A | 10.7 | N/A | N/A |
| Number of children/adolescents testing HIV+ through DHT (pre-intervention) or bPITC (post-intervention) at OPDs | 10.5 | 9.7 | 0.7574 | -7.9% |
| Number of children/adolescents tested HIV+ in hospitals (ART clinics +OPDs) | 10.5 | 20.3 | 0.0256 | 93.7% |
| Number of children/adolescents enrolled on ART through tPITC at ART clinics | N/A | 5.8 | N/A | N/A |
| Number of children/adolescents enrolled on ART through DHT (pre-intervention) or bPITC (post-intervention) at OPDs | 7.3 | 6.3 | 0.5819 | -13.6% |
| Number of children/adolescents enrolled on ART in the hospitals (ART clinics + OPDs) | 7.3 | 12.2 | 0.0388 | 65.9% |

DHT: diagnostic HIV testing; tPITC: targeted provider-initiated counselling and testing; bPITC: blanket provider-initiated testing and counselling; ART: antiretroviral therapy; OPD: outpatient department; Not applicable because activity not implemented during the pre-intervention period. **p value comparing the monthly mean outcome of pre-intervention vs. post-intervention period.
